# Routine HIV Testing in Adolescents and Young Adults Presenting to an Outpatient Clinic in Durban, South Africa

**DOI:** 10.1371/journal.pone.0045507

**Published:** 2012-09-20

**Authors:** Lynn Ramirez-Avila, Kristy Nixon, Farzad Noubary, Janet Giddy, Elena Losina, Rochelle P. Walensky, Ingrid V. Bassett

**Affiliations:** 1 Division of Infectious Diseases, Children’s Hospital Boston, Boston, Massachusetts, United States of America; 2 Medical Practice Evaluation Center, Massachusetts General Hospital, Boston, Massachusetts, United States of America; 3 McCord Hospital, Durban, KwaZulu-Natal, South Africa; 4 Division of General Medicine, Massachusetts General Hospital, Boston, Massachusetts, United States of America; 5 Department of Orthopedic Surgery, Brigham and Women’s Hospital, Boston, Massachusetts, United States of America; 6 Division of Rheumatology, Brigham and Women’s Hospital, Boston, Massachusetts, United States of America; 7 Department of Biostatistics, Boston University, Boston, Massachusetts, United States of America; 8 Center for AIDS Research, Harvard Medical School, Cambridge, Massachusetts, United States of America; 9 Division of Infectious Diseases, Massachusetts General Hospital, Boston, Massachusetts, United States of America; 10 Division of Infectious Disease, Brigham and Women’s Hospital, Boston, Massachusetts, United States of America; London School of Hygiene and Tropical Medicine, United Kingdom

## Abstract

**Objectives:**

Although youth (12–24 years) in Sub-Saharan Africa have a high HIV risk, many have poor access to HIV testing services and are unaware of their status. Our objective was to evaluate the proportion of adolescents (12–17 years) and young adults (18–24 years) who underwent HIV testing and the prevalence among those tested in an urban adult outpatient clinic with a routine HIV testing program in Durban, South Africa.

**Design:**

We conducted a retrospective cross-sectional analysis of adolescent and young adult outpatient records between February 2008 and December 2009.

**Methods:**

We determined the number of unique outpatient visitors, HIV tests, and positive rapid tests among those tested.

**Results:**

During the study period, 956 adolescents registered in the outpatient clinic, of which 527 (55%) were female. Among adolescents, 260/527 (49%, 95% CI 45–54%) females underwent HIV testing compared to 129/429 (30%, 95% CI 26–35%) males (p<0.01). The HIV prevalence among the 389 (41%, 95% CI 38–44%) adolescents who underwent testing was 16% (95% CI 13–20%) and did not vary by gender (p = 0.99). During this period, there were 2,351 young adult registrations, and of these 1,492 (63%) were female. The proportion consenting for HIV testing was similar among females 980/1,492 (66%, 95% CI 63–68%) and males 543/859 (63%, 95% CI 60–66%, p = 0.25). Among the 1,523 (65%, 95% CI 63–67%) young adults who underwent testing, the HIV prevalence was 22% (95% CI 19–24%) in females versus 14% in males (95% CI 11–17%, p<0.01).

**Conclusions:**

Although the HIV prevalence is high among youth participating in an adult outpatient clinic routine HIV program, the uptake of testing is low, especially among 12–17 year old males. There is an urgent need to offer targeted, age-appropriate routine HIV testing to youth presenting to outpatient clinics in epidemic settings.

## Introduction

Youth (12–24 years) are disproportionately affected by the HIV epidemic in sub-Saharan Africa. [1], [2] In South Africa, the HIV prevalence rapidly rises through adolescence with an HIV prevalence of 3% among 2–14 year olds, 15% among 15–24 year olds, and 24% among those ≥25 year olds. [3] Despite the high HIV prevalence, youth testing rates are low. [4], [5] Compared to 47% of South Africans adults who report ever having an HIV test, [6] only 25% of females and 15% of males 15–24 years old report prior HIV testing [7].

In 2007 the World Health Organization (WHO) recommended routine HIV testing among youth in epidemic settings, and South Africa implemented an HIV testing initiative that proposed to mobilize youth and to ensure ‘youth friendly’ HIV testing services –that is services and programs specifically designed for this population. [6], [8] Unfortunately, little guidance on the operationalization of youth routine testing, which refers to offering HIV testing as a standard component of medical care, irrespective of signs or symptoms, is provided in these recommendations.

To better understand testing in an urban adult outpatient clinic with a routine HIV testing program for patients ≥12 years, we evaluated the number of adolescents (12–17 years) and young adults (18–24 years) who had HIV testing and the prevalence among those tested in Durban, South Africa. We hypothesized that adolescent and young adult females would have higher HIV testing uptake and prevalence compared to adolescent and young adult males [3], [4].

## Methods

This retrospective cross-sectional analysis was completed at McCord Hospital, an urban medical center in Kwa-Zulu Natal, the province with the highest HIV prevalence in South Africa. [9] The overall estimated HIV prevalence in Kwa-Zulu Natal is 25%, but is higher than 39% among women attending antenatal clinic. [9] McCord is a government-subsidized, semi-private facility that predominantly serves an urban population from the greater Durban area. [10] McCord charges a subsidized fee for all service and treatment and has a 142-bed inpatient ward and a well-established HIV clinic. [10] McCord also has a general medical outpatient clinic with separate adult and pediatric services that serves 3,000–4,000 predominantly Zulu-speaking adults per month. [10], [11] The outpatient clinic provides a range of services including primary health, urgent care, and emergency care for trauma and critically ill patients; approximately 60% of patients present for episodic care or for a single visit. [11] McCord has an antenatal program that is separate from the outpatient clinic. During the study period, patients paid an outpatient clinic consultation fee (ZAR 280 = US $40).

Patients ≥12 years are managed in the adult outpatient clinic. Since 2008, the outpatient clinic has had a routine HIV testing program and, per hospital guidelines, patients ≥12 years who are not known to be HIV-infected should be offered routine HIV testing on an opt-out basis at no additional charge. [11] Per South African law, patients ≥12 years can consent for HIV testing without guardian approval. [12] Prior to physician consultation, dedicated HIV counselors obtained consent, pre- and post-test counseling, and two concurrent rapid blood fingerprick HIV tests. Newly-diagnosed HIV-infected patients were referred for CD4 testing at no additional fee and HIV clinic registration. HIV clinic services were subsidized by the United States President’s Emergency Plan for AIDS Relief (PEPFAR) and the KwaZulu-Natal Department of Health. [10] Patients paid a subsidized monthly fee for HIV clinic services [10].

For this analysis, we defined youth as those 12–24 years, adolescents those 12–17 years, and young adults those 18–24 years. We reviewed outpatient clinic records between February 2008 and December 2009 and determined the number of unique outpatient visitors, HIV tests, and positive rapid HIV tests in adolescents (12–17 years) and young adults (18–24 years) when an HIV counselor available (Monday-Saturday, 7∶00 am-4∶30 pm). The study outcomes were the proportion that underwent an HIV test and the HIV prevalence among adolescents and young adults who underwent testing, stratified by gender. We used the Z-test to compare proportions and the Cochran-Armitage test for trend to assess changes in both the proportion of patients tested and the HIV prevalence among those tested by age. All analyses were carried out using R software (R version 2.11.1). Ethics approval was obtained from the McCord Hospital, Children’s Hospital Boston and Partners HealthCare Human Research Committees. Given that the number of clinic registrations, HIV tests, and positive rapid HIV tests were collected for clinic evaluation purposes and analyzed in aggregate, individual patient consent was not obtained. To our knowledge patients/guardians do not consent to have their records used for research purposes at the time of clinic registration. All data were analyzed in aggregate and anonymously.

## Results

From February 2008 to December 2009, there were 956 adolescent registrants (12–17 years) and of these 527 (55%) were female (Table 1). Overall, 389 (41%) underwent HIV testing. Of the adolescent females, 260/527 (49%, 95% CI 45–54%) underwent HIV testing versus 129/429 (30%, 95% CI 26–35%) adolescent males (p<0.01). The HIV prevalence among both male and female adolescents who underwent testing was 16% (95% CI 13–20%, p = 0.99).

**Table 1:** The number and percentage of youth who underwent HIV testing and were HIV-infected in an outpatient clinic in Durban, South Africa.

	Total (%, 96% CI)	Females (%, 95% CI)	Males (%, 95% CI)		p-value
**Adolescent (12–17 years)**					
Total	956 (100)	527 (55, 52–58%)		429 (45, 42–48%)	
HIV tested	389 (41, 38–44%)	260 (49, 45–54%)		129 (30, 26–35%)	<0.01
HIV-infected among those tested	62 (16, 13–20%)	42 (16, 12–21%)		20 (16, 10–23%)	0.99
**Young Adult (18–24 years)**					
Total	2,351 (100)	1,492 (63, 61–65%)		859 (37, 35–39%)	
HIV tested	1,523 (65, 63–67%)	980 (66, 63–68%)		543 (63, 60–66%)	0.25
HIV-infected among those tested	288 (19, 17–21%)	213 (22, 19–24%)		75 (14, 11–17%)	<0.01

The HIV testing and HIV-infected percentages represent column percentages.

During the same period, 2,351 young adults (18–24 years) registered in the outpatient clinic and of these 1,492 (63%) were female (Table 1). Of the young adult females, 980/1,492 (66%, 95% CI 63–68%) were HIV tested compared to 543/859 (63%, 95% CI 60–66%) young adult males (p = 0.25). The HIV prevalence among young adults who underwent testing was 19% (95% CI 17–21%). The HIV prevalence was significantly higher among young adult females who underwent testing (22%, 95% CI 19–24%) compared to young adult males who underwent testing (14%, 95% CI 11–17%, p<0.01).

**Figure 1:**
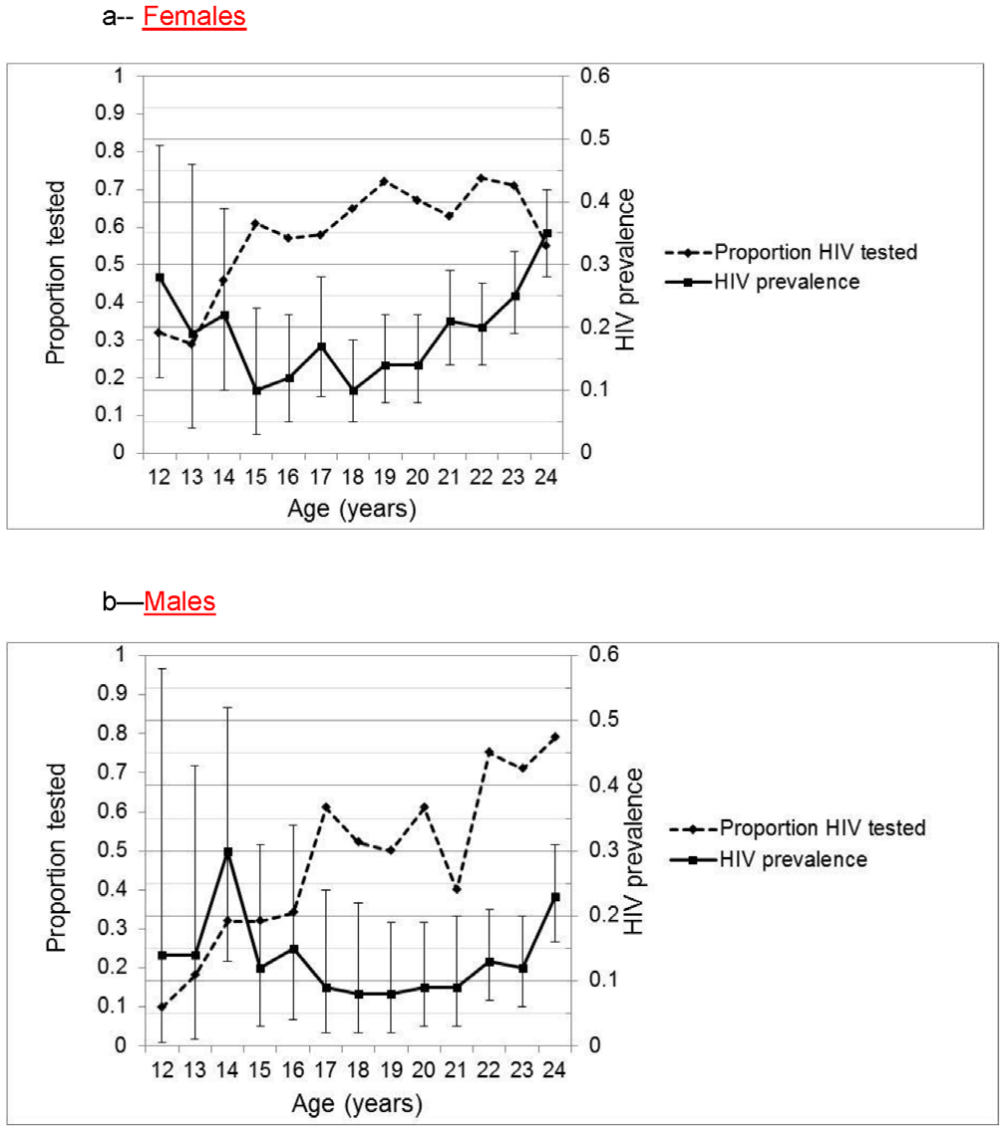
Proportion of females and males who underwent testing and the HIV prevalence among those tested, by age. The dashed line represents the proportion who underwent HIV testing by age. The solid line is the HIV prevalence among those tested with error bars representing 95% confidence intervals. Figure 1a–Females. Figure 1b–Males.

HIV testing was lowest among adolescents and steadily increased among young adults for each gender (Figure 1a and 1b, dashed line). Overall, only 21% of presenting 12 year olds were HIV tested compared to 63% of 24 year olds (data not shown). The Cochran-Armitage test for trend revealed a significant increase in the proportion of patients who completed HIV testing with increasing age for females and males (p<0.01). The testing among 12-year-old males was particularly low, with only 10% (7/72) of males compared to 32% (25/78) of females completing HIV testing (Figure 1a and 1b, dashed line). The low HIV testing rate was in contrast to the high HIV prevalence, among those who were tested, and this discrepancy held across all age groups for each gender (Figure 1a and 1b, solid line, error bars represent 95% confidence intervals). The HIV prevalence gradually increased through young adulthood for each gender (solid line). The Cochran-Armitage test for trend revealed a significant increase in the HIV prevalence among females that were tested with increasing age (p<0.01).

## Discussion

We evaluated the proportion of adolescents and young adults who had an HIV test and the prevalence among those tested in an adult outpatient clinic with a routine HIV testing program in Durban, South Africa. Although the HIV prevalence was 16% among adolescents and 19% among young adults who underwent testing, there was a large gap in HIV testing, particularly among the adolescent males (12–17 years).

Despite recent studies that have shown that HIV testing may be an efficacious strategy for identifying HIV-infected youth in the primary clinic setting, [13] we observed low HIV testing among adolescents in an outpatient routine testing program. Although the proportion of youth who had HIV testing in our routine adult testing program increased with age, only 21% of 12 year olds underwent testing. The low HIV testing among the adolescents could have resulted from multiple individual, caretaker, social/cultural, clinic, and community barriers. On an individual level, youth may decline HIV testing because they do not consider themselves at risk for infection because they are not sexually active, or because they are asymptomatic. [14], [15], [16] The perceived association between sexual debut and HIV risk is especially concerning for the subset of adolescents who have late diagnosis of perinatally-acquired infection. [15] Caretakers and healthcare workers can also limit HIV testing opportunities if they do not think the youth is at risk for HIV infection. [15] Uncertainty about the legal age of consent for HIV testing and the informed consent process, [17] especially for youth presenting with caretakers, can also be barriers. Additional barriers to youth testing include concern for confidentiality, stigma of using a testing facility, unfriendly clinic staff, and that South African youth ≥12 years are regarded as adults and managed in adult healthcare programs [16], [18], [19], [20].

HIV testing for adolescent females in this outpatient routine HIV testing program was higher than for adolescent males. Previous studies have shown the higher overall testing rates among females in Sub-Saharan Africa. [3], [21], [22], [23] In particular, female youth in Kenya [14] and South Africa [4], [5], [7] have also been reported to have higher HIV testing rates than male youth. The difference in HIV testing by gender is thought to occur because female youth are more likely to be offered testing, [14] in part due to their participation in antenatal programs. [5] The high HIV testing among young adult females in our outpatient clinic study is not due to antenatal testing which is a separate program at our study site. The gender differential in HIV testing in our study highlights the importance of targeting routine testing programs specifically to adolescent males, who are reported to have a lower median age of sexual debut and higher number of partners than adolescent females [4].

The low uptake of routine HIV testing among youth represents a missed opportunity for testing and undermines current South African efforts to extend counseling and testing to this population that experiences a high burden of new HIV infections. [1], [2], [6], [16], [20], [24] Testing services that are designed for youth are urgently needed to increase testing uptake. [1], [2], [16], [20], [24] The WHO framework for youth-friendly health services includes programs that are accessible, equitable, acceptable, appropriate, comprehensive, effective, and efficient for young people. [25] In practice, youth friendly testing services, should be easily accessible to young people by offering appropriate hours and free services, staffed by providers who are motivated and trained to meet the needs of youth, and involve youth in the development of services. [13], [16], [20], [24], [26], [27], [28], [29] In addition to being the entry point to HIV treatment, youth HIV testing services are valuable to provide HIV prevention counseling, [20], [30] promote regular patterns of HIV testing in adulthood for HIV-negative youth, and impact the dramatic rise in HIV incidence in transition to adulthood.

Understanding the barriers to testing uptake, developing youth-friendly HIV testing services, and optimizing the delivery of youth HIV testing is challenging. Involving South African youth in research to inform HIV testing programs is crucial yet requires parental consent for those ≤18 years, which can be difficult to obtain and limits participation. [2], [24], [31], [32], [33] Although there are no specific youth HIV testing studies, designing a standardized youth HIV testing program seems impractical because of the developmental heterogeneity of this population. [19], [20], [34] Optimizing the venue for youth HIV diagnostic services, such as school-based or mobile unit testing, may improve youth testing opportunities. [20], [35], [36] Also, the need for dedicated youth health care professionals, clinics, and units, including HIV services, requires further attention in resource-limited settings [19], [20].

The HIV prevalence among adolescents and young adults participating in this outpatient routine HIV testing program was high. As reported in national data, the HIV prevalence rose steadily and disproportionately among young adult females. [3], [4] The high HIV prevalence among young adult females as compared to young adult males has been attributed to a range of factors including differences in biological susceptibility, higher rates of intergenerational sex, and lower use of condoms. [37], [38], [39] As compared to the young adults, the HIV prevalence among adolescents did not differ by gender. It is possible that the similar adolescent prevalence across gender is reflective of late progressors who acquired HIV perinatally. [15] In our analysis, the rise in HIV prevalence among those tested begins at 16–17 years which corresponds to the median age of sexual debut in South Africa [4].

This analysis is based on cross-sectional clinic data and has several limitations. The study site is not representative of the large volume primary healthcare clinics where care is free and the HIV testing uptake and prevalence would likely be different. Although routine HIV testing for patients >12 years is clinic policy, we cannot determine if all the youth who presented were in fact offered HIV testing. The retrospective and aggregate nature of the study precludes analyzing the individual factors associated with routine HIV testing among youth. For example, we do not know the reason the youth presented to the clinic, if they presented with their caretaker, if they refused HIV testing, or if they had a CD4 count and enrolled in continuity HIV services for those who were newly-diagnosed HIV-infected. It is possible that HIV testing was preferentially offered by healthcare workers or requested by youth with a high suspicion for infection. We cannot assess the impact of the caretaker nor structural clinic barriers on routine youth HIV testing.

Given the large rise and high HIV prevalence among youth, optimizing HIV testing is essential for effective treatment and prevention and tackling the HIV epidemic in this population. There is an urgent need to offer directed, comprehensive, and age-appropriate HIV testing to youth presenting to outpatient clinics in epidemic settings in order to increase HIV knowledge, encourage regular HIV testing, and diagnose and link to care those who are HIV-infected.
